# Image Enhanced Mask R-CNN: A Deep Learning Pipeline with New Evaluation Measures for Wind Turbine Blade Defect Detection and Classification

**DOI:** 10.3390/jimaging7030046

**Published:** 2021-03-04

**Authors:** Jiajun Zhang, Georgina Cosma, Jason Watkins

**Affiliations:** 1 Department of Computer Science, School of Science, Loughborough University, Loughborough LE11 3TT, UK; 2 Railston & Co. Ltd., Nottingham NG7 2TU, UK; jason@railstons.com

**Keywords:** defect detection, wind turbine blade, deep learning, convolutional neural network, region-based convolutional neural networks, evaluation measure, mask R-CNN, YOLOv3, YOLOv4

## Abstract

Demand for wind power has grown, and this has increased wind turbine blade (WTB) inspections and defect repairs. This paper empirically investigates the performance of state-of-the-art deep learning algorithms, namely, YOLOv3, YOLOv4, and Mask R-CNN for detecting and classifying defects by type. The paper proposes new performance evaluation measures suitable for defect detection tasks, and these are: Prediction Box Accuracy, Recognition Rate, and False Label Rate. Experiments were carried out using a dataset, provided by the industrial partner, that contains images from WTB inspections. Three variations of the dataset were constructed using different image augmentation settings. Results of the experiments revealed that on average, across all proposed evaluation measures, Mask R-CNN outperformed all other algorithms when transformation-based augmentations (i.e., rotation and flipping) were applied. In particular, when using the best dataset, the mean Weighted Average (mWA) values (i.e., mWA is the average of the proposed measures) achieved were: Mask R-CNN: 86.74%, YOLOv3: 70.08%, and YOLOv4: 78.28%. The paper also proposes a new defect detection pipeline, called Image Enhanced Mask R-CNN (IE Mask R-CNN), that includes the best combination of image enhancement and augmentation techniques for pre-processing the dataset, and a Mask R-CNN model tuned for the task of WTB defect detection and classification.

## 1. Introduction

Demand for wind power has grown, and this has led to an increase in the manufacturing of wind turbines, which in turn has resulted in an increase in wind turbine blade (WTB) inspections and repairs. Defect detection systems can be utilised to inspect the regular operation of WTBs. The operation efficiency of wind turbines can be reduced if defects exist on the surface of blades [1]. Most inspection processes require engineers to carry out manual examinations and repairs, and such tasks can be hazardous since most wind turbines are massive in size and installed in high-speed wind areas. Non-Destructive Testing (NDT) is a commonly adopted testing technique that evaluates the properties of WTBs for defects, without causing damage to the WTBs. Currently, many NDT techniques are utilised to detect defects on WTB surfaces in industries. For example, Lockin and Infrared Thermography techniques to monitor the surface health of material [2,3]; a visual testing system to monitor defects using fixed cameras [4]; acoustic emission test data to check the structural health of WTBs [5]; and microwave imaging to detect delamination [6].

Recently, vision-based techniques have received attention for defect detection applications, and these techniques use cameras (or drones) and Deep Learning (DL) algorithms to analyse captured images/videos to locate the defected areas [7,8]. Reddy et al. [9] proposed a Convolutional Neural Network (CNN) to recognise the presence of cracks on the surfaces of WTBs with an accuracy of 94.94%. Yang et al. [10] proposed a ResNet50 model to identify multi-type WTB defects and achieved 95% classification accuracy, but their dataset was imbalanced since only 10% of the images had defects. Deng et al. [11] trained YOLOv2 for defect detection and found that YOLOv2 outperformed the Faster R-CNN, each achieving 77% and 74.5%, respectively. However, YOLOv2 is now an outdated version (released in 2016) since the current version is YOLOv4.

Jia et al. [12] evaluated the effect of different augmentation methods and found that applying transformation-based augmentations which used cropping, flipping and rotation, increased accuracy by 1.0–3.5% compared to the original dataset. Applying specific image enhancement techniques (e.g., white balance, contrast enhancement, and greyscale) to highlight the features of areas with defects, and image augmentation techniques (e.g., flipping and rotation) based on geometric transformations of images can improve the detection performance of the DL detection models [13,14]. Furthermore, transforming images to greyscale could reduce the noise, enhance the defect features and increase a model’s detection performance [15]. With greyscale images, the models only learn the contrast values rather than the RGB values in an image and this may result in faster training times.

YOLOv3 [16], YOLOv4 [17] and Mask R-CNN [18] are state-of-art DL-based object detection algorithms. DL algorithms have not fully exploited for the task of WTB defect detection, and defect detection in general. This paper presents an empirical comparison of the detection performance of DL algorithms, namely, Mask R-CNN, YOLOv3, and YOLOv4, when tuned for the defect detection task and when using various image augmentation and enhancement techniques. The paper presents a novel defect detection pipeline based on the Mask R-CNN algorithm and which includes an empirically selected set of image enhancement and augmentation techniques. Furthermore, traditional evaluation measures of Recall, Precision and $F_{1}$-score, do not provide a holistic overview of the defect detection performance of DL detection models. Therefore, this paper proposes new evaluation measures, namely Prediction Box Accuracy (PBA), Recognition Rate (RR), and False Label Rate (FLR), suitable for the task of defect detection. Traditional measures were contextualised for the task, and thereafter the traditional and proposed evaluation measures were adopted for comparing the performance of DL algorithms.

This paper is organised as follows. Section 2 provides a discussion on DL based defect detection methods. Section 3 describes the experiment methodology that includes a discussion of the dataset that was provided by Railston & Co. Ltd.; describes various image augmentation techniques that were applied to the dataset to empirically determine the best combination of techniques for the task; and proposes three new evaluation measures in addition to contextualised traditional performance evaluation measures for defect detection. Section 4 presents the results and analysis of experiments with YOLOv3, YOLOv4, and Mask R-CNN for the task of WTB defect detection when using four datasets, i.e., the original dataset plus three datasets that were constructed using a combination of image augmentation techniques. Section 4 also presents the results of experiments to determine whether image enhancement can further improve the defect detection performance of the Mask R-CNN model. This section also presents a proposed defect detection pipeline, namely the Image Enhanced Mask R-CNN, that was developed using the best performing network, i.e., Mask R-CNN, and an empirically selected combination of image augmentation and enhancement techniques. Section 5 provides a discussion, conclusion, and suggestions for future work.

## 2. Related Methods

This section describes relevant literature that focuses on DL-based defect detection. Machine learning (ML) and DL algorithms have been applied to detect defects on surfaces. For example, neural network and Bayesian network algorithms were proposed to fuse sensor data for detecting defects in apples [19]; multi-resolution decomposition and neural networks have been applied to detect and classify defects on textile fabrics [20,21].

Literature discussing defect detection methods using ML is limited. In 2004, Graham et al. [22] designed a neural network to examine damages of carbon fibre composites, and this is one of the earliest works on defect detection using neural networks. Graham et al. did not present a quantitative analysis of the neural network’s performance. Although their experiments and results were limited, they found that the neural network algorithm could recognise damaged areas. In 2014, Soukup and Huber-Mörk [23] proposed an algorithm for detecting cracks on the steel surfaces. Soukup and Huber-Mörk’s algorithm combined a DL algorithm, namely the CNN, with a model-based algorithm which utilised specular reflection and diffuse scattering techniques to identify defects. Their results revealed that the detection error rate decreased by 6.55% when using CNN instead of the model-based algorithm. Soukup and Huber-Mork [23] also highlighted that their proposed CNN algorithm could distinguish the types of surface defects if the detection model was trained with a quality dataset. In 2018, Wang et al. [24] applied ResNet CNN and ML algorithms (i.e., support vector machine (SVM), linear regression (LR), random forest (RF) and bagging) to detect defects on blueberry fruit. Their results showed that CNN algorithms achieved an accuracy of 88.44% and an Area Under the Curve (AUC) of 92.48%. CNN outperformed other ML algorithms by reaching 8–20% higher accuracy.

In 2019, Narayanan [25] applied SVM and CNN algorithms for the task of defect detection in fused filament fabrication. The SVM required 65% less training time than the CNN model, but its recall rate was 8.07% lower than that of the CNN model. Wang et al. [26] proposed a DL-based CNN to inspect the product defects, and compared the detection performance with an ML approach that utilised the Histogram of Oriented Gradient feature (HOG) technique and an SVM model. Their results illustrated that the CNN achieved an accuracy of 99.60%, whereas the ML model achieved an accuracy of 93.07%. However, CNN’s detection speed was slower than the ML model by 24.30 ms.

With regards to WTB defect detection, NDT and ML techniques were utilised for identifying surface defects of WTBs, and DL algorithms were employed to analyse the outputs. For example, in 1999, Kawiecki [27] used a simple neural network to identify defects by analysing the collected data. In 2009, Jasinien et al. [28], utilised ultrasonic and radiographic techniques to detect defects. In 2015, Protopapadakis and Doulamis [29] proposed a CNN-based algorithm to detect cracks on tunnel surfaces with 88.6% detection accuracy, that was higher than conventional ML algorithms, i.e., SVM’s accuracy reached 71.6%; k-nearest neighbour model’s accuracy reached 74.6%, and the classification tree model’s accuracy reached 67.3%.

DL techniques have been utilised to detect defects in images. In 2017, Yu et al. [30] proposed an image-based damage recognition approach to identify defects on WTB surfaces. They composed a framework for defect detection comprising a CNN and an SVM. The framework was trained using the ImageNet Large Scale Visual Recognition Challenge (ILSVRC) dataset [31], and experimental results showed that their proposed method reached 100% accuracy. Yu et al.’s defect detection system used a two-stage method. The first stage utilised a CNN for extracting defect features from input images and for locating the defects. In the second stage, an SVM model was utilised for classifying the defects by type. Yu et al.’s experiment results showed that the two-stage structure is promising for identifying defects by analysing images that were captured from inspection cameras, and that a large enough training dataset is essential for achieving high detection accuracy.

In 2019, Qiu et al. [32] designed a WTB detection system, YSODA, which is based on the YOLOv3 algorithm. Qiu et al. modified the YOLO architecture to support the multi-scale feature pyramid in the layers of CNN. They captured 4000 images of WTB defects using a camera that was embedded in a drone. These images were then augmented with different processes, such as flip, rotation, blur, and zoom, and this resulted in 23,807 images that were utilised for training the model. YSODA outperformed the original YOLO algorithm, especially for small-sized defect detection. YSODA achieved 91.3% accuracy with 24 fps detection speed, and YOLOv3 only achieved 88.7% accuracy with 30 fps. Although YSODA outperformed YOLOv3, YSODA’s speed was slower than the original YOLOv3 algorithm because the complexity of feature recognition had increased. In 2019, Shihavuddin et al. [33], exploited the faster R-CNN algorithm to detect multiple defect types of WTBs. In this experiment, Shihavuddin et al. applied various data augmentation approaches, such as flip, blur and contrast normalisation, to enhance the training data and improve detection accuracy. Their proposed methods achieved 81.10% mAP@IoU(0.5) (detection performance) with the 2.11 s detection speed. Table 1 provides a summary on current WTB defect detection techniques that use DL and ML algorithms.

## 3. Materials and Methods

This section describes the dataset and image augmentation techniques applied to the dataset (see Section 3.1). It describes the traditional (see Section 3.2) and proposed measures (see Section 3.3) for evaluating the defect detection performance of the YOLOv3 [16], YOLOv4 [17], and Mask R-CNN [18] algorithms with and without image augmentation methods. The experiment methodology is described in Section 3.4.

### 3.1. Dataset and Image Augmentation

The dataset used for the experiments was provided by the industrial partner Railston & Co. Ltd. The dataset comprises images that were captured by engineers during manual WTB inspections. The engineers labelled the images into four categories: crack, erosion, void and ‘other’ defects. The original size of each captured image is 2592 × 1936 pixels. All images were uniformly cropped and resized to 1920 × 1080 pixels with 16:9 ratio. The number of images for each defect type are shown in Table 2.

Image augmentation techniques were applied to the original dataset (Dataset D0). Dataset D0 consists of 191 images classified into four types of defects. Note that the ‘other’ defect type contains delamination and debonding defects, and these were combined into one type named ‘other’ because there were only 22 images that belonged to those defects.

Datasets D1–D3 were created using different combinations of image augmentation techniques (e.g., flipping, rotation, and greyscale) can enhance the detection performance of DL methods [30,32,33]. Influenced by literature, three combinations of augmentation techniques were devised and applied to the original dataset (D0) to create three new datasets (Dataset 1 (D1), Dataset 2 (D2), and Dataset 3 (D3)) as shown in Table 3. Image augmentation artificially expands the size of a dataset by creating modified versions of the images found in the dataset using techniques such as greyscale, flip and rotation. The DL detection model was then trained on the original dataset (i.e., D0), and thereafter on each of the three datasets (i.e., D1–D3).

### 3.2. Traditional Performance Evaluation Measures for Defect Detection

Traditional evaluation measures were based on Precision (as shown in (1)), Recall (as shown in (2)), the $F_{1}$-score (as shown in (3)) and mAP@IoU. These evaluation measures are described in the context of defect detection. The contextualised concepts of TP, FP and FN are provided below.

- True Positive (TP) predictions—a defect area that is correctly detected and classified by the model.
- False Positive (FP) predictions—an area that has been incorrectly identified as a defect. There are two types of FPs. (1) The predicted area does not overlap with a labelled area; and (2) the predicted area is overlapping with a labelled area, but the defect’s type is misclassified.
- False Negative (FN) predictions—a labelled area that has not been detected by the model.

(1) $$\mathrm{Detection}\;\mathrm{Precision}=\frac{\mathrm{Total}\;\mathrm{TP}\;\mathrm{Predictions}}{\mathrm{Total}\;\mathrm{TP}\;\mathrm{Predictions}+\mathrm{Total}\;\mathrm{FP}\;\mathrm{Predictions}}$$

(2) $$\mathrm{Detection}\;\mathrm{Recall}=\frac{\mathrm{Total}\;\mathrm{TP}\;\mathrm{Predictions}}{\mathrm{Total}\;\mathrm{TP}\;\mathrm{Predictions}+\mathrm{Total}\;\mathrm{FN}\;\mathrm{Predictions}}$$

(3) $$\mathrm{Detection}\;F_{1}-\mathrm{score}=2\times \frac{\mathrm{Detection}\;\mathrm{Precision}\times \mathrm{Detection}\;\mathrm{Recall}}{\mathrm{Detection}\;\mathrm{Precision}+\mathrm{Detection}\;\mathrm{Recall}}$$

The mean Average Prevision (mAP) at Intersection over Union (IoU), mAP@IoU, is a measure commonly adopted for evaluating the performance of DL detection models for machine vision tasks. mAP@IoU was also adopted during the evaluations. The mean Average Precision (mAP) shown in (4) [34], is the average AP over all classes.

(4) $$\mathrm{mAP}=\frac{1}{C}\sum_{i=1}^{C}AP_{i}$$

where $AP_{i}$ is the AP value for the i-th class and *C* is the total number of classes (i.e., defect types) being classified. A prediction whose bounding box IoU value is greater than a threshold is considered as a TP, otherwise, the prediction is considered as an FP. An IoU threshold value of 0.5 is commonly used to indicate the average detection performance. In the experiments described in this paper, the threshold for IoU was set to 0.5.

### 3.3. Proposed Performance Evaluation Measures for Defect Detection

**Figure 1:**
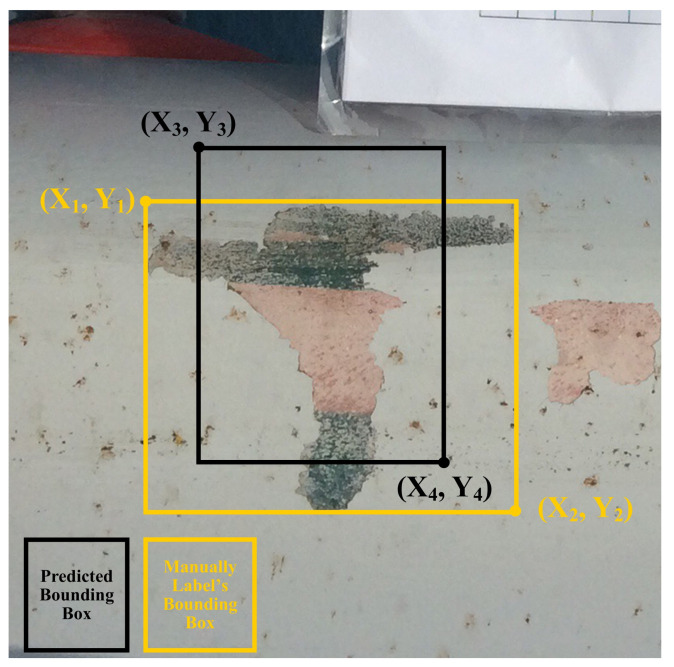
Bounding Box Accuracy—yellow box shows the bounding box of the manually labelled defect, and the black box is an example predicted bounding box that may be generated by the detection model. BBA computes the difference between two overlapping bounding boxes by calculating the area between the overlapping boxes. If there is no overlap between the bounding boxes, the BBA value will be 0.

Let bounding box accuracy (BBA) be the measure of the performance of a detection model in terms of how accurately it predicts the defect’s bounding box compared to the label’s bounding box, as shown in (5) and illustrated in Figure 1.

(5) $$\mathrm{Bounding}\;\mathrm{Box}\;\mathrm{Accuracy}=\left\{\begin{array}{cc} \frac{\mathrm{WidthAcc}+\mathrm{HeightAcc}}{2}, & \mathrm{if}\;((\mathrm{WidthAcc}>0)\wedge (\mathrm{HeightAcc}>0))\\ 0 & \mathrm{otherwise} \end{array}\right.$$

where WidthAcc and HeightAcc is Width Accuracy and Height Accuracy, respectively, which are calculated using (6) and (7) shown below.

(6) $$\mathrm{Width}\;\mathrm{Accuracy}=1-\frac{|x_{1}-x_{3}|+|x_{2}-x_{4}|}{|max\left(x_{2},x_{4}\right)-min\left(x_{1},x_{3}\right)|}$$

where $x_{1}$, $x_{2}$, $x_{3}$ and $x_{4}$ are the values of the *x* coordinate points shown in Figure 1.

(7) $$\mathrm{Height}\;\mathrm{Accuracy}=1-\frac{|y_{1}-y_{3}|+|y_{2}-y_{4}|}{|max\left(y_{2},y_{4}\right)-min\left(y_{1},y_{3}\right)|}$$

where $y_{1}$, $y_{2}$, $y_{3}$ and $y_{4}$ are the values of the *y* coordinate points shown in Figure 1.

If the predicted bounding box does not overlap with the label’s bounding box or the prediction is FN, the BBA value will be 0. If the bounding boxes overlap, then BBA will be a positive value indicating the bounding box difference. The BBA value is 1 when the bounding box of a predicted defect area is perfectly overlapping with the label’s bounding box, and hence the difference is 0. The three new evaluation measures proposed for the task of defect detection are described below and these measures utilise BBA.

#### 3.3.1. Prediction Box Accuracy

Prediction Box Accuracy (PBA) calculates the average BBA of all BBA values greater than 0, as shown in (8). PBA, computes the average degree of overlap (i.e., BBA) between the labelled and the predicted boxes of the defects that have been identified by the model.

(8) $$\mathrm{PBA}=\begin{array}{c} \frac{1}{n}\sum_{i=1}^{n}BBA_{i} \end{array}$$

where *i* is the index of each prediction, and *n* is the total number of predictions.

#### 3.3.2. Recognition Rate

Recognition Rate (RR) measures the recognition performance of a detection model. RR, as shown in (9), calculates the proportion of defects that were recognised as defects over all known defects, without taking into consideration the defect type classification results. If a defect is correctly detected but its type is incorrectly classified, it will be counted in the RR.

(9) $$\mathrm{RR}=\begin{array}{cc} \frac{1}{N}\sum_{i=1}^{n}1, & \mathrm{if}\;BBA_{i}>0 \end{array}$$

where *i* is the index of each prediction, *n* is the total number of predictions with a BBA value greater than 0, and *N* is the total number of the labelled defects.

#### 3.3.3. False Label Rate

False Label Rate (FLR), as shown in (10) computes the proportion of the predictions with a false label (i.e., $\mathrm{Predicted}\;{\mathrm{Type}}_{i}$ ≠ $\mathrm{Labelled}\;{\mathrm{Type}}_{i}$) and whose bounding box has an overlap with the manual label (i.e., BBA value > 0). Hence, FLR is the ratio of the total number of misclassified predictions that have overlapping bounding boxes over the total number of predictions with overlapping bounding boxes.

(10) $$\begin{array}{cc} \mathrm{FLR}=\frac{1}{N}\sum_{i=1}^{n}1, & \mathrm{if}\;\left({\mathrm{BBA}}_{i}>0\right)\wedge \left(\mathrm{Predicted}\;{\mathrm{Type}}_{i}\neq \mathrm{Labelled}\;{\mathrm{Type}}_{i}\right) \end{array}$$

where *i* is the index of each prediction, *n* is the total number of predictions with a BBA value > 0 and a false label (i.e., $\mathrm{Predicted}\;{\mathrm{Type}}_{i}$ ≠ $\mathrm{Labelled}\;{\mathrm{Type}}_{i}$), and *N* is the total number of predictions with BBA values > 0.

### 3.4. Experimental Setup

The datasets used for the experiments are described in Section 3.1. Areas with defects were annotated using the VGG Image Annotator (VIA) [35] tool. The process of image annotation creates a set of annotations (a.k.a labels) that DL detection models use during the training process to learn areas of interest with better accuracy. The annotation formats required for YOLOv3, YOLOv4, and Mask R-CNN are different, and thus the annotations were converted to the appropriate format for each model. Each model requires a set of inputs: (1) a set of images; and (2) a file containing annotations of defects in the required format. Different augmentation strategies were applied to the original dataset to derive new datasets that can be utilised to identify the best image augmentation strategies for defect detection using DL algorithms. Applying various augmentation strategies resulted in four datasets (see Table 3) and each dataset was split into a train and test set with an 80:20% ratio. The number of images distributed across Datasets D0–D3 is shown in Table 4.

During the training process, each model provides a loss value that indicates the overall learning progress. The loss value is low when the model learns the defect features. Therefore, by default, the training process stops when the training loss value converges and it is lower than each algorithm’s default settings (YOLO: 0.06, Mask R-CNN: 0.08). At the end of the training process, the model generates a weight file to perform the defect detection; every weight stores the feature map, containing the defect’s features. Finally, the performance of a trained model is evaluated using a test set that has not been previously seen by the model (i.e., it was unseen during the training process). The experiments were performed using a high-end desktop computer equipped with an i7 CPU, RTX 2070 GPU, and 64 GB RAM.

## 4. Results

**Figure 2:**
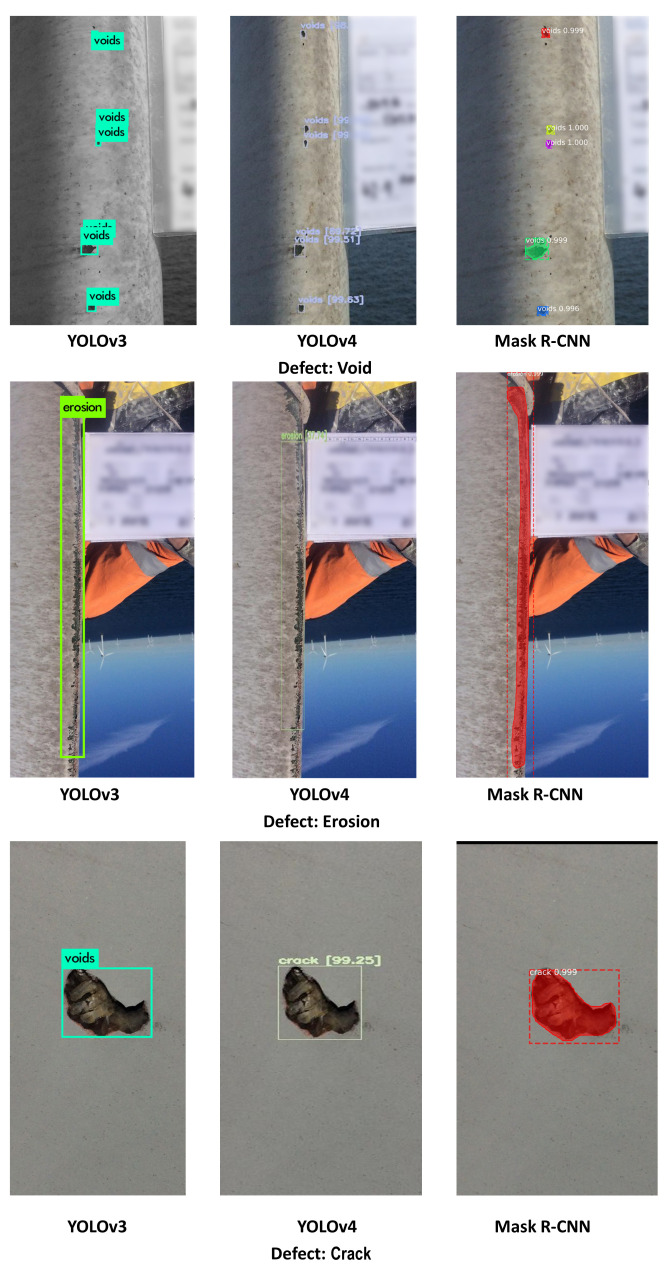
Example outputs of DL algorithms. The figure shows three outputs of YOLOv3, YOLOv4 and Mask R-CNN. All algorithms recognised the defects, however, YOLOv3 incorrectly classified a crack defect as a void defect; and the prediction boxes did not comprehensively cover the large-sized defect area, such as erosion defect, in YOLOv4.

This section describes the results of the experiments with YOLOv3, YOLOv4, and Mask R-CNN for the task of WTB defect detection through using four different datasets (i.e., Datasets D0-D3), where each dataset was constructed using a combination of image augmentation techniques (see Table 3). Contextualised traditional and proposed measures described in Section 3.2 and Section 3.3 were adopted to evaluate the performance of the models. As an example, Figure 2 shows three outputs of each algorithm.

Table 5, Table 6 and Table 7 provide the performance evaluation results of the YOLOv3, YOLOv4, and Mask R-CNN, respectively. In these tables, the weighted average (WA) value, as shown in (11), provides the overall performance for each model.

(11) $$\mathrm{WA}=\begin{array}{c} \frac{1}{N}\sum_{i=1}^{t}V_{i}\times D_{i} \end{array}$$

where WA is the weighted average value, *N* is the total number of labelled defects, *t* is the total number of defect types, $V_{i}$ is the evaluation measure result for defect type *i*, and $D_{i}$ is the total number of labelled defects belonging to defect type *i*.

### 4.1. Performance Evaluation of YOLOv3, YOLOv4, and Mask R-CNN

This subsection describes the results of the experiments carried out to evaluate the performance of YOLOv3, YOLOv4, and Mask R-CNN for WTB defect detection when using the test set described in Section 3.1.

**YOLOv3:** The performance evaluation results of YOLOv3 are shown in Table 5. The results revealed that the best model was YOLOv3(D3), and reached the highest mWA of 70.08% ± 0.15. Although the WA(FLR) values of YOLOv3(D1) and YOLOv3(D2) were lower than those of YOLOv3(D3) by 2.9% and 6.1% respectively, their WA(RR)s were also lower than those of YOLO(D3) by 9.24% and 19.58%, respectively. Regarding the average performance of YOLOv3, the mStd was the highest for YOLOv3(D3), i.e., ±0.15, which indicates that the model was less stable than when trained using other datasets. However, the relatively high mStd value was mainly because the YOLOv3(D3) model performed worse on detecting crack defects compared to other defects.

**YOLOv4:** The performance evaluation results of YOLOv4 are shown in Table 6. Observing the results, it appears that YOLOv4’s performance was best with Dataset D1 and Dataset D2. YOLOv4(D1) reached the highest mAW (78.28%), a relatively low mStd ± 0.20 value, and the highest WA(RR) (79.11%) and these results indicate that it is the better model. YOLOv4(D2) reached higher WA(PBA) and WA$F_{1}$-score values than YOLOv4(D1), however, the WA(RR) of YOLOv4(D1) was much higher, i.e., 5.94%, than that of YOLOv4(D2). These results suggest that Dataset D1 is the better dataset to train YOLOv4.

**Mask R-CNN:** The performance evaluation results of Mask R-CNN are shown in Table 7. The Average Performance results show that Mask R-CNN(D2) returned the best model, outperforming other Mask R-CNN models. Observing the Average Performance results, Mask R-CNN(D2) reached the highest mAW and mAP@IoU(0.5) values, i.e., a mAW value of 86.74%, and a mAP@IoU(0.5) of 82.57%. Mask R-CNN(D2) also achieved the lowest mStd (±0.05) value. Furthermore, with regards to detecting defect types, Mask R-CNN(D2) achieved the highest performance for all except for the void type, where Mask R-CNN(D3) slightly outperformed Mask RCNN(D2) by 0.25%.

### 4.2. Comparison of YOLOv3, YOLOv4 and Mask R-CNN

Table 8 presents a comparison of the performance of the best models that resulted from Section 4.1. The models under comparison are: Mask R-CNN(D2), YOLOv3(D3), and YOLOv4(D1).

**Mask R-CNN(D2) Compared to YOLOv3(D3):**

**Figure 3:**
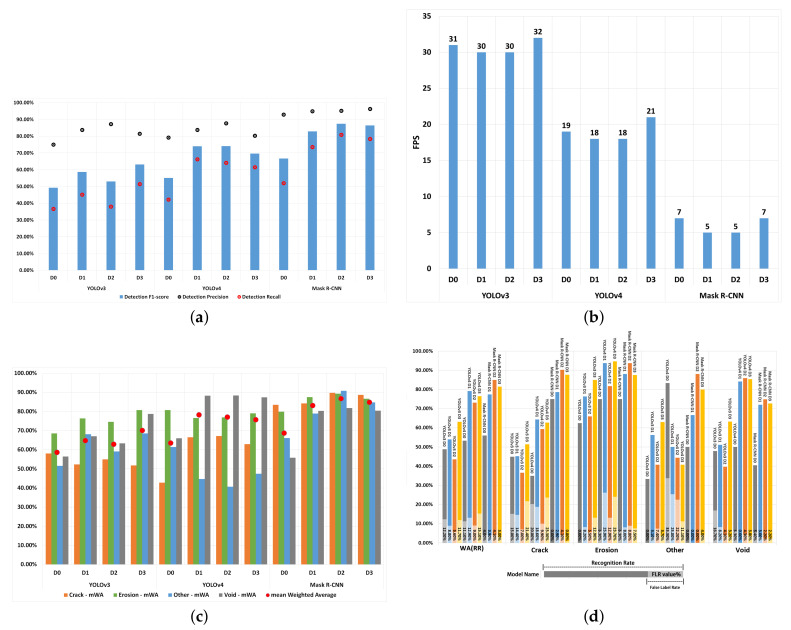
Performance Evaluation Diagrams for YOLOv3, YOLOv4 and Mask R-CNN. (**a**) Traditional Performance Evaluation. (**b**) Detection Speed Evaluation. (**c**) mean Weighted Average Performance Evaluation. (**d**) RR and FLR Evaluation.

Mask R-CNN outperformed YOLOv3 and YOLOv4 in terms of PBA performance, detecting all except the void defect types. With regards to detecting voids, YOLOv3 and YOLOv4 outperformed Mask R-CNN by achieving PBA values that are 11.84% and 2.33%, respectively, (as shown in Table 8). This may be due to the fact that void defects are usually small in size (and smaller than the other defect types). Figure 3d illustrates that Mask R-CNN is relatively weaker than YOLOv3 and YOLOv4 algorithms in recognising small-sized defects (i.e., void).

Regarding the RR (see Figure 3d) and $F_{1}$-score (see Figure 3a) results, Mask R-CNN(D2) outperformed YOLOv3(D3) across all defect types. Table 8 shows that, on average, mAP@IoU(0.5) was higher by 29.29%, WA(RR) was higher by 21.79%, WA(FLR) was lower by 7.60%, and WA($F_{1}$) was higher by 24.37% when using Mask R-CNN compared to YOLOv3. Finally, looking at the Average Performance results (see Figure 3c), Mask R-CNN outperformed YOLOv3 when considering all evaluation measures.

**Mask R-CNN(D2) compared to YOLOv4(D1):** In Table 8, the PBA of YOLOv4 was 2.33% higher than Mask R-CNN in detecting void defects. The std(PBA) of Mask R-CNN was lower than that of YOLOV4 by 0.151 points and WA(PBA) was higher by 6.09%, and these values indicate that the Mask R-CNN is relatively more stable and accurate in BBA predictions than YOLOv4. On average, Mask R-CNN outperformed YOLOv4 with regards to WA(RR) by 5.87%. The WA(FLR) and std(RR) values of Mask R-CNN were on average lower than those of YOLOv4 by 8.80% and 0.117, respectively. This suggests that the Mask R-CNN is more stable than YOLOv4 in detecting defects by type. However, due to Mask R-CNN’s weak ability in detecting small-sized defects, the RR was 9.27% lower in void defect detection, as also shown in Figure 3d. In overall, Table 8 shows that Mask R-CNN outperformed YOLOv4 with a 8.47% higher value for mAW, 26.99% higher mAP@IoU(0.5) and a 8.80% lower FLR.

**YOLOv4(D1) compared to YOLOv3(D3):** On Average YOLOv4 returned a 8.19% higher mAW value, but compared to YOLOv3, it also returned higher mStd and mFLR values, by, 0.05 and 1.20% respectively (as shown in Table 8), which are indicators of worse performance. A closer look at the performance of YOLOv4 and YOLOv3 with regards to their ability in detecting individual defect types, YOLOv3 outperformed YOLOv4 with regards to WA(PBA) performance by 2.27% (see Figure 3c) and an std(PBA) difference of 0.02 (see Table 8), whereas YOLOV4 outperformed YOLOv3 by 15.93% with regards to WA(RR), as shown in Figure 3d. Table 8 illustrates that YOLOv4’s WA($F_{1}$) score was 10.90% higher than that of YOLOv3.

**Comparison of detection speed:** The detection speed values of each algorithm are shown in Figure 3b. Mask R-CNN’s detection speed is much slower than that of YOLOv3 and YOLOv4 by 25 and 13 fps, respectively. However, due to the high and stable detection performance of the Mask R-CNN model, it is still regarded as the most suitable detection model for detecting defects. In real-time inspection tasks, detection speeds of 20 and 30 fps would be too fast since the engineers would not be able to respond to the outputs of the model at those speeds. Therefore, the detection speed of a DL algorithm will need to be tuned to match its real-world use.

### 4.3. An Investigation into Whether Image Enhancement Can Further Improve the Results of the Mask R-CNN Model

**Figure 4:**
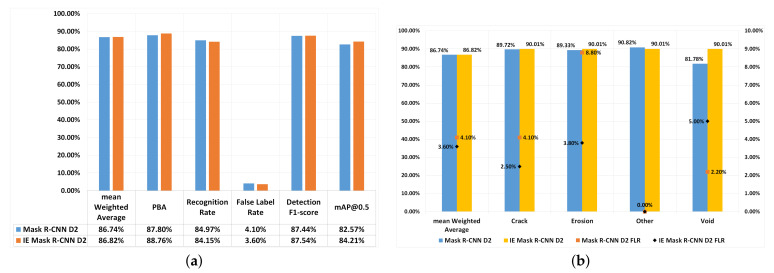
Performance comparison between Mask R-CNN(D2) and IE Mask R-CNN(D2). (**a**) Overall performance comparisons. (**b**) mWA evaluation and FLR comparisons. Left axis is used for mWA, and the right axis is used for FLR.

Based on the experiments carried out thus far, as described in Section 4, Mask R-CNN(D2) was the best performing model. This section describes the results of experiments that apply image enhancement techniques to the dataset. Initially, image enhancement techniques, namely white balance (WB) and adaptive histogram equalisation (AHE) were applied to the original dataset. After that, image augmentation techniques are applied to the image enhanced dataset. These image augmentation techniques are those provided in Table 3 Dataset D2. This detection pipeline is called Image Enhanced Mask R-CNN (IE Mask R-CNN). Figure 4 shows IE Mask R-CNN’s performance, and the results are compared with Mask R-CNN(D2).

In overall, the performance of IE Mask R-CNN(D2) (mAW:86.82%, $F_{1}$-score: 87.44%) and Mask R-CNN(D2) (mAW: 86.74%, $F_{1}$-score: 87.54 %) were close, as shown in Figure 4a. With IE Mask R-CNN(D2) mAP@IoU(0.5) was 1.64% higher, PBA was 0.94% higher and FLR was 0.5% lower than Mask R-CNN(D2). The higher PBA values suggest an overall improvement in defect detection and bounding box prediction when applying image enhancement techniques (i.e., WB and AHE).

Figure 4b compares the mWA and FLR of each defect type for IE Mask R-CNN(D2) and Mask R-CNN(D2). The figure shows that the mWA value of defect type ‘other’ was lower by 0.81% compared to IE Mask R-CNN(D2), whereas the mWA values of all other defect types were higher (i.e., by 0.29% for crack, by 0.68% for erosion, and by 8.23% for void) compared to Mask R-CNN(D2). The fact that there was no improvement in detecting the ‘other’ type of defects when using the IE Mask R-CNN(D2) compared to Mask R-CNN(D2) is likely to be because the class ‘other’ only had 22 images in the original dataset, and therefore the models were not able to learn all the features of the defects of type ‘other’ due to complex defect data and lack of training data.

The FLRs for crack and erosion were decreased by 1.6% and 5% respectively when IE Mask R-CNN was using, but the FLR of the void defect was higher than Mask R-CNN by 2.8%. Considering the size of each defect type, the void defects were relatively smaller than crack and erosion. Given that IE Mask R-CNN(D2) outperformed Mask R-CNN(D2) with regards to detecting the larger-sized defects (i.e., erosion and crack) suggests that image enhancement techniques can reduce the number of misclassified images that contain the larger sized defects.

### 4.4. IE Mask R-CNN: Proposed Deep Learning Defect Detection Pipeline

**Figure 5:**
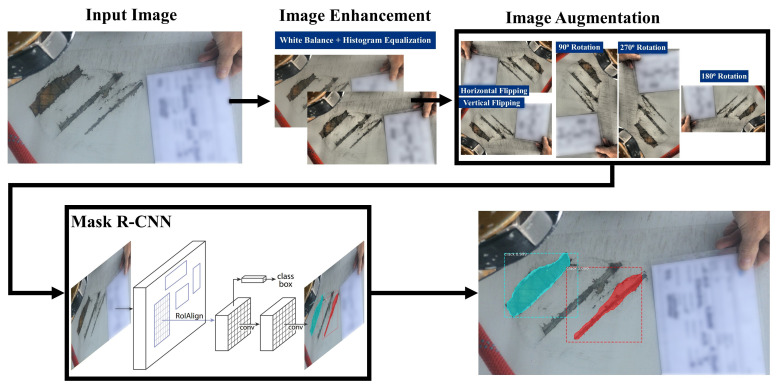
IE Mask R-CNN: The proposed Image Enhanced Mask R-CNN pipeline.

Based on the results of the experiments discussed in Section 4, a DL model for defect detection is proposed. The pipeline structure of IE Mask R-CNN is shown in Figure 5. This section describes the components of the proposed IE Mask R-CNN pipeline.

**Input images:** The input images are the original images captured by engineers during inspections, such as those discussed in Section 3.1. When new batches of images along with their annotations become available, these can be used for re-training the model, as a strategy for improving its performance. The images are required to be in JPG or PNG-format and need to be at least 400 pixels in height and width dimensions and they can be in any aspect ratio.

**Image enhancement:** Image enhancement and augmentation methods are initially applied to the dataset, and thereafter the dataset is trained using a Mask R-CNN algorithm. The architecture of the Mask R-CNN algorithm is described below. Image enhancement techniques include WB [36] and AHE [37,38]. WB normalises the images such that they have the same temperature. The IE Mask R-CNN pipeline utilises a WB image pre-processing tool developed by Afifi and Brown [39] that can automatically adjust the image temperatures. The IE Mask R-CNN pipeline includes the AHE technique which utilises a contrast enhancement algorithm implemented by Pizer et al. [38] to distinguish the areas whose colour is significantly different across the image set.

**Image augmentation:** In Section 4, Mask R-CNN achieved the best performance with Dataset D2, and thus the image augmentation techniques used in Dataset D2 were included in the IE Mask R-CNN pipeline. Details of the image augmentation techniques that were fused to derive Dataset D2 are provided in Table 3.

**Figure 6:**
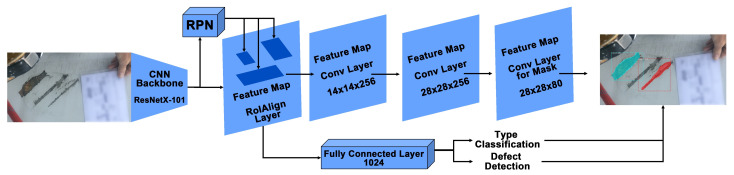
Mask R-CNN architecture with ResNetX-101 backbone and Fully Connected layers (FC layers). This architecture was embedded in the proposed IE Mask R-CNN pipeline.

**Mask R-CNN algorithm:** Mask R-CNN [18] is the detection algorithm that is used in the proposed pipeline. The architecture of Mask R-CNN is shown in Figure 6. In the first stage, the pre-processed images are trained using a ResNetX-101 CNN backbone [40], and a region proposal network that generates a RoIAlign feature map that stores feature information of defects. In the second stage, a fully connected layer detects and classifies the detected defects. Moreover, additional convolutional layers learn the masked areas of the predicted defect areas.

## 5. Discussion and Conclusions

This paper investigates the performance of DL algorithms for the task of WTB defect detection and classification, and proposes a new Mask R-CNN based pipeline for the task. A dataset of images captured by engineers during manual WTB inspections was provided by the industrial partner Railston & Co. Ltd. The engineers labelled the images into four categories: crack, erosion, void, and ‘other’ defects. The main contributions of the paper are summarised as follows:

The paper investigates the impact of various image augmentation and image enhancement techniques on the performance of DL algorithms for the task of WTB defect detection. The original dataset was transformed three times using a different set of image augmentation techniques. As a result, experiments were carried out using four datasets (i.e., original dataset and 3 datasets derived after applying transformation techniques). Empirical evaluations were carried out with the original and augmented datasets to investigate the performance of state-of-the-art DL algorithms, namely, YOLOv3, YOLOv4, and Mask R-CNN for detecting defect areas (i.e., bounding boxes around detected areas) and for classifying the detected defects by type.

Traditional evaluation measures of Recall, Precision and $F_{1}$-score, do not provide a holistic overview of the defect detection performance of DL detection models. Therefore, this paper proposes new evaluation measures, namely Prediction Box Accuracy (PBA), Recognition Rate (RR), and False Label Rate (FLR). The proposed measures consider the bounding box accuracy of the detected defect areas and were designed for the task of evaluating the performance of DL detection models applied to defect detection tasks. Furthermore, the traditional evaluation measures of Precision, Recall, and $F_{1}$-score were contextualised for the task of defect detection.

The contextualised traditional and proposed evaluation measures were adopted for comparing the performance of the DL detection models. The results of the experiments revealed that on average, across all evaluation measures (i.e., mean Weighted Average (mWA)), Mask R-CNN outperformed other DL algorithms when transformation-based augmentations (i.e., rotation and flipping) were applied to the image dataset. Mask R-CNN outperformed YOLOv3 and YOLOv4, and achieved the highest detection performance with mAP@IoU(0.5): 82.57%, mAW: 86.74%, PBA: 87.80%, RR: 84.97% and FLR: 4.1%. This paper proposes a new defect detection pipeline, called IE Mask R-CNN, which applies image enhancement and augmentation methods. IE Mask R-CNN reached mAP@IoU(0.5): 84.21%, mWA: 86.82%, PBA: 88.76% and FLR: 3.6%, and outperformed Mask R-CNN in mAP@IoU(0.5) (by 1.64%), mAW (by 0.08%), PBA (by 0.94%) and FLR (by 0.5%).

In future work, additional image enhancement techniques that can highlight the colour of defect areas from the images will be explored. Dataset re-sampling methods will also be empirically evaluated to improve the balance between images across the defect types. In Mask R-CNN, many CNN parameters can be adjusted for different detection purposes, such as anchor-scale, Region of Interest number, and backbone stride. These parameters can be further investigated to provide the best setting for different detection situations. The condition monitoring and fault diagnosis of WTBs deserve further investigation through using the IE Mask R-CNN. A monitoring system can be designed to define the potential WTB health problems beforehand and deliver the engineers to execute checking and repairing programs. Since demand for wind power has grown, this has resulted in an increase in the manufacturing, inspection, and repairs of wind turbines and their blades. The operation efficiency of wind turbines is affected by defects that exist on the surface of blades. Defect detection systems based on ML and DL methods have been utilised to inspect the regular operation of WTBs damage diagnosis [41,42] and condition monitoring [43,44,45]. Future work includes extending the proposed pipeline for the task of condition monitoring. Research on the topic of defect detection and especially WTB defect detection with DL algorithms is still at an early stage. The proposed Image Enhanced Mask R-CNN pipeline is suitable for the task of WTB defect detection and can be applied to other surfaces. Therefore, future work also includes evaluating the performance of IE Mask R-CNN for other tasks such as train wheel defect detection.

## Figures and Tables

**Table 1:** Summary of DL and ML techniques for WTB defect detection and classification.

Author	Year	Method	Result	Limitation
Kawiecki [27]	1999	Neural Network	<15% test error	Data collection requires professional NDT techniques. CNN architecture is outdated.
Jasinien et al. [28]	2009	Ultrasonic & radiographic	N/A	Requires professional NDT. Paper lacks a thorough evaluation and only provides example outputs.
Protopapadakis & Doulamis [29]	2015	CNN, SVM, k-NN, DT	CNN: 88.6%, SVM: 71.6%, k-NN: 74.6%, DT: 67.3%	N/A.
Yu et al. [30]	2017	CNN+SVM	100% Accuracy	Methods can only classify the defects but cannot provide location information of the defect in the images.
Qiu et al. [32]	2019	YSODA (CNN)	91.3% Accuracy	Detection speed is slower than YOLOv3
Shihavuddin et al. [33]	2019	Faster R-CNN	81.10% mAP@IoU(0.5)	Slow detection speed.
Reddy et al. [9]	2019	CNN	94.94% Accuracy	Method only achieved high accuracy in binary classification mode (fault vs. non-fault).
Yang et al. [10]	2020	CNN	95.58% Accuracy	Long training time.
Deng et al. [11]	2020	YOLOv2 (CNN)	77% mAP@IoU(0.5)	Outdated YOLO version. Slow detection speed.

**Table 2:** Number of images per defect type in the original dataset. The original dataset is the baseline dataset.

Type	Number of Images
Crack	55
Erosion	62
Void	52
Other	22
Total	191

**Table 3:** Image augmentation settings of each dataset.

Dataset	Image Augmentation Settings
D0	Original
D1	Original
	+Vertical Flip + Horizontal Flip
	+90${}^{\circ }$ Rotation + 180${}^{\circ }$ Rotation + 270${}^{\circ }$ Rotation
	+Greyscale Original
D2	Original
	+Vertical Flip + Horizontal Flip
	+90${}^{\circ }$ Rotation + 180${}^{\circ }$ Rotation + 270${}^{\circ }$ Rotation
D3	Greyscale Original
	+Greyscale Vertical Flip + Greyscale Horizontal Flip
	+Greyscale 90${}^{\circ }$ Rotation + Greyscale 180${}^{\circ }$ Rotation + Greyscale 270${}^{\circ }$ Rotation

**Table 4:** Number of training and testing images in Datasets D0–D3. The last column shows the total number of images of each dataset.

Dataset	Number of Training Images	Number of Testing Images	Total Images
D0	147	44	191
D1	1069	268	1337
D2	923	223	1146
D3	923	223	1146

**Table 5:** YOLOv3: Performance evaluation on test dataset. WA is the weighted average as defined in (11). mWA is the mean WA of PBA, RR, and Detection $F_{1}$-score. mStd is the mean std of PBA, RR, and Detection $F_{1}$-score. mFLR is the weighted average of FLR across all defect types.

Prediction Box Accuracy (PBA)					
Defect type		Dataset D0	Dataset D1	Dataset D2	Dataset D3
Crack		**87.88% ± 0.11**	69.20% ± 0.10	84.83% ± 0.076	64.29% ± 0.23
Erosion		66.06% ± 0.24	75.35% ± 0.083	**84.79% ± 0.080**	79.20% ± 0.091
Void		79.10% ± 0.17	80.10% ± 0.097	**99.32% ± 0.052**	99.22% ± 0.051
Other		71.49% ± 0.10	**92.76% ± 0.098**	89.03% ± 0.074	70.04% ± 0.094
	std(PBA)	±0.09	±0.10	±**0.069**	±0.15
	WA(PBA)	77.59% ± 0.19	81.62% ± 0.10	**91.99% ± 0.07**	83.98% ± 0.13
Recognition Rate (RR) and (False Label Rate (FLR))					
Defect type		Dataset D0	Dataset D1	Dataset D2	Dataset D3
Cracks		45.00% (15.0%)	45.21% (14.4%)	36.63% **(7.0%)**	**51.45%** (21.4%)
Erosion		62.50% **(0.0%)**	76.36% (8.2%)	65.93% (5.5%)	**84.95%** (12.9%)
Void		47.92% (16.7%)	51.14% (8.2%)	39.69% (4.1%)	**63.24% (3.2%)**
Other		33.33% **(0.0%)**	53.16% (3.1%)	40.74% (7.4%)	**62.96%** (3.7%)
	std(RR)	**±0.12**	±0.13	±0.14	±0.14
	WA(RR)	48.89% (12.2%)	53.94% (8.8%)	43.60% **(5.6%)**	**63.18%** (11.7%)
Detection $F_{1}$-score					
Defect type		Dataset D0	Dataset D1	Dataset D2	Dataset D3
Crack		41.38%	42.49%	**43.40%**	39.69%
Erosion		76.92%	77.32%	72.85%	**77.91%**
Void		42.25%	57.31%	50.92%	**73.51%**
Other		50.00%	68.00%	47.37%	**72.73%**
	std($F_{1}$)	±0.17	±0.15	**±0.13**	±0.17
	WA($F_{1}$)	49.25%	58.67%	52.95%	**63.08%**
Average Performance					
Defect type		Dataset D0	Dataset D1	Dataset D2	Dataset D3
Crack		58.09%	52.30%	**60.73%**	51.81%
Erosion		68.49%	76.34%	74.52%	**80.69%**
Void		56.42%	62.85%	63.31%	**78.66%**
Other		51.61%	**71.31%**	56.58%	67.34%
	mAP@IoU(0.5)	37.10%	**55.69%**	49.66%	53.28%
	mStd	±0.13	±0.13	**±0.11**	±0.15
	mWA	58.58%	64.74%	62.58%	**70.08%**
	mFLR	12.2%	8.8%	**5.6%**	11.7%

**Table 6:** YOLOv4: Performance evaluation on test dataset. WA is the weighted average as defined in (11). mWA is the mean WA of PBA, RR, and Detection $F_{1}$-score. mStd is the mean std of PBA, RR, and Detection $F_{1}$-score. mFLR is the weighted average of FLR across all defect types.

Prediction Box Accuracy					
Defect type		Dataset D0	Dataset D1	Dataset D2	Dataset D3
Crack		70.99% ± 0.25	79.50% ± 0.082	**80.08% ± 0.11**	77.47% ± 0.16
Erosion		**88.45% ± 0.37**	65.95% ± 0.20	73.27% ± 0.18	69.51% ± 0.15
Void		89.55% ± 0.45	89.71% ± 0.14	**91.33% ± 0.092**	88.76% ± 0.097
Other		46.48% ± 0.30	50.60% ± 0.24	46.65% ± 0.34	**59.64% ± 0.17**
	std(PBA)	±0.20	±0.17	±0.19	**±0.12**
	WA(PBA)	82.08% ± 0.23	81.71% ± 0.16	**84.08% ± 0.14**	80.83% ± 0.14
Recognition Rate (RR) and (False Label Rate (FLR))					
Defect type		Dataset D0	Dataset D1	Dataset D2	Dataset D3
Crack		35.00% (20.0%)	**64.36%** (18.6%)	59.30% **(9.9%)**	62.79% (23.3%)
Erosion		75.00% **(6.3%)**	93.75% (25.9%)	81.72% (12.9%)	**94.62%** (23.7%)
Void		50.00% (6.3%)	84.09% **(0.0%)**	**85.95%** (4.3%)	85.41% (3.8%)
Other		**83.33%** (33.3%)	50.00% (25.0%)	44.44% (22.2%)	40.74% **(11.1%)**
	std(RR)	±0.22	**±0.20**	**±0.20**	±0.24
	WA(RR)	53.33% (11.1%)	**79.11%** (12.9%)	73.17% **(9.0%)**	76.52% (15.1%)
Detection *F*1-score					
Defect type		Dataset D0	Dataset D1	Dataset D2	Dataset D3
Crack		22.22%	55.66%	**62.04%**	48.57%
Erosion		**78.57%**	70.05%	75.74%	72.93%
Void		58.33%	**90.90%**	87.79%	88.05%
Other		**54.55%**	33.33%	30.77%	42.11%
	std($F_{1}$)	±0.23	±0.24	±0.25	**±0.21**
	WA($F_{1}$)	55.07%	73.98%	**74.09%**	69.60%
Average Performance (type classification)					
Defect type		Dataset D0	Dataset D1	Dataset D2	Dataset D3
Crack		42.74%	66.51%	**67.14%**	62.94%
Erosion		**80.67%**	76.58%	76.91%	79.02%
Void		65.96%	88.23%	**88.36%**	87.41%
Other		**61.45%**	44.64%	40.62%	47.50%
	mAP@IoU(0.5)	39.55%	55.58%	**56.53%**	53.67%
	mStd	±0.22	±0.20	±0.22	**±0.19**
	mWA	63.49%	**78.28%**	77.11%	75.65%
	mFLR	11.1%	12.9%	**9.0%**	15.1%

**Table 7:** Mask R-CNN: Performance evaluation on test dataset. WA is the weighted average as defined in (11). mWA is the mean WA of PBA, RR, and Detection $F_{1}$-score. mStd is the mean std of PBA, RR, and Detection $F_{1}$-score. mFLR is the weighted average of FLR across all defect types.

Prediction Box Accuracy					
Defect type		Dataset D0	Dataset D1	Dataset D2	Dataset D3
Crack		89.64% ± 0.37	**89.05% ± 0.15**	88.49% ± 0.15	85.81% ± 0.17
Erosion		86.17% ± 0.069	**89.39% ± 0.18**	86.50% ± 0.21	87.02% ± 0.21
Void		76.99% ± 0.23	**88.66% ± 0.11**	87.38% ± 0.087	86.77% ± 0.083
Other		81.64% ± 0.12	89.94% ± 0.045	**90.84% ± 0.049**	89.70% ± 0.087
	std(PBA)	±0.055	±0.054	±0.019	±**0.017**
	WA(PBA)	83.56% ± 0.15	**89.05% ± 0.14**	87.80% ± 0.14	86.68% ± 0.15
Recognition Rate (RR) and (False Label Rate (FLR))					
Defect type		Dataset D0	Dataset D1	Dataset D2	Dataset D3
Crack		75.00% **(0.0%)**	78.68% (2.9%)	**90.16%** (4.1%)	87.70% (0.8%)
Erosion		75.00% **(6.3%)**	88.00% (8.0%)	**93.75%** (8.8%)	87.50% (7.5%)
Void		40.54% (5.4%)	72.02% (3.0%)	**74.82% (2.2%)**	72.66% **(2.2%)**
Other		50.00% **(0.0%)**	66.67% **(0.0%)**	**88.00% (0.0%)**	80.00% (4.0%)
	std(RR)	±0.18	±0.092	±0.083	**±0.072**
	WA(RR)	56.00% (4.0%)	77.42% (3.9%)	**84.97%** (4.1%)	81.42% **(3.0%)**
Detection *F*1-score					
Defect type		Dataset D0	Dataset D1	Dataset D2	Dataset D3
Crack		85.71%	84.77%	90.52%	**92.58%**
Erosion		78.57%	85.11%	**87.74%**	85.33%
Void		50.00%	80.28%	**83.13%**	81.67%
Other		66.67%	80.00%	**93.62%**	84.44%
	std($F_{1}$)	±0.16	**±0.028**	±0.045	±0.047
	WA($F_{1}$)	66.67%	82.86%	**87.44%**	86.45%
Average Performance					
Defect type		Dataset D0	Dataset D1	Dataset D2	Dataset D3
Crack		83.45%	84.17%	**89.72%**	88.70%
Erosion		79.91%	87.50%	**89.33%**	86.62%
Void		55.84%	80.32%	81.78%	**82.03%**
Other		66.10%	78.87%	**90.82%**	84.72%
	mAP@IoU(0.5)	57.47%	77.53%	**82.57%**	76.80%
	mStd	±0.13	±0.06	**±0.05**	**±0.05**
	mWA	68.74%	83.11%	**86.74%**	84.85%
	mFLR	4.0%	3.9%	4.1%	**3.0%**

**Table 8:** Performance evaluation comparison of Mask R-CNN(D2), YOLOv3(D3), and YOLOv4(D1).

Prediction Box Accuracy (PBA)				
Defect type		Mask R-CNN vs. YOLOv3	Mask R-CNN vs. YOLOv4	YOLOv4 vs. YOLOv3
Crack		+24.20%	+8.99%	+15.21%
Erosion		+7.29%	+20.54%	−13.25%
Void		−11.84%	−2.33%	−9.51%
Other		+20.81%	+40.24%	−19.44%
	std(PBA)	−0.131	−0.151	+0.02
	WA(PBA)	+3.82%	+6.09%	−2.27%
Recognition Rate (RR) and (False Label Rate (FLR))				
Defect type		Mask R-CNN vs. YOLOv3	Mask R-CNN vs. YOLOv4	YOLOv4 vs. YOLOv3
Crack		+38.72% (−17.30%)	+25.80% (−14.50%)	+12.92% (−2.80%)
Erosion		+8.80% (−4.10%)	+0.00% (−17.10%)	+8.80% (+13.00%)
Void		+11.58% (−1.00%)	−9.27% (+2.20%)	+20.85% (−3.20%)
Other		+25.04% (−3.70%)	+38.00% (−25.00%)	−12.96% (+21.30%)
	std(RR)	−0.057	−0.117	0.06
	WA(RR)	+21.79% (−7.60%)	+5.87% (−8.80%)	+15.93% (+1.20%)
Detection $F_{1}$-score				
Defect type		Mask R-CNN vs. YOLOv3	Mask R-CNN vs. YOLOv4	YOLOv4 vs. YOLOv3
Crack		+50.82%	+34.85%	+15.97%
Erosion		+9.83%	+17.70%	−7.86%
Void		+9.62%	−7.78%	+17.40%
Other		+20.89%	+60.28%	−39.39%
	std($F_{1}$)	−0.125	−0.195	+0.07
	WA($F_{1}$)	+24.37%	+13.47%	+10.90%
Average Performance				
Defect type		Mask R-CNN vs. YOLOv3	Mask R-CNN vs. YOLOv4	YOLOv4 vs. YOLOv3
Crack		+37.91%	+23.22%	+14.70%
Erosion		+8.64%	+12.75%	−4.10%
Void		+3.12%	−6.46%	+9.58%
Other		+22.25%	+46.18%	−23.93%
	mAP@IoU(0.5)	29.29%	26.99%	2.30%
	mStd	−0.104	−0.154	+0.05
	mAW	+16.66%	+8.47%	+8.19%
	mFLR	−7.60%	−8.80%	+1.20%

## Data Availability

Not Applicable.

## Abbreviations

The following abbreviations are used in this manuscript:

WTB	Wind Turbine Blade
NDT	Non-Destructive Testing
DL	Deep Learning
CNN	Convolutional Neural Network
YOLO	You Only Look Once
YOLOv2	YOLO version 2
YOLOv3	YOLO version 3
YOLOv4	YOLO version 4
Mask R-CNN	Mask Region-based Convolutional Neural Network
mAP	Mean Average Precision
PBA	Prediction Box Accuracy
RR	Recognition Rate
FLR	False Label Rate
IE Mask R-CNN	Image Enhanced Mask R-CNN
ML	Machine Learning
SVM	Support Vector Machine
LR	Linear Regression
RF	Random Forest
AUC	Area Under the Curve
HOG	Histogram of Oriented Gradient feature
ILSVRC	ImageNet Large Scale Visual Recognition Challenge
k-NN	k-Nearest Neighbour
DT	Decision Tree
D0	Dataset D0
D1	Dataset D1
D2	Dataset D2
D3	Dataset D3
TP	True Positive
FP	False Positive
FN	False Negative
IoU	Intersection over Union
BBA	Bounding Box Accuracy
WidthA	Width Accuracy
HeightA	Height Accuracy
VIA	VGG Image Annotator
WA	Weighted Average
mWA	mean Weighted Average
std	Standard Deviation
WB	White Balance
AHE	Adaptive Histogram Equalisation
